# Verses

**Published:** 1907-05

**Authors:** 


### VERSES.

### GENTLE SPRING THOUGHTS.

The violet will shortly blow,
The crocus lift its cup;
And in the fields the hemp will grow
To string the umpire up.

—New York Sun.

### TRUE HAPPINESS.

'Tis not in money nor in land
That life its happiness reveals;
It is dodging microbes and
Assimilating three square meals.

—Washington Star.

### OH-HEIGH-OH!

"No," said his wife, "I will not need
A bonnet, not this spring,
Nor a new dress, nor any gloves,
In fact, not anything;
And I won't care to go away
And leave the city smoke
And dust and grime"—then some one knocked,
And the man awoke.

—Houston Post.

### REMEDY WANTED.

Wouldn't be much grumblin'
Anywhere, I guess,
And but little weepin'
Or unhappiness,
If our wives would only
Find some way to quit
Frettin' over troubles
That ain't struck 'em yit.

—Chicago Record Herald.

### DEATH.

Death enters and there's no defense;
His time there's none can tell;
He'll in a moment call thee hence
To heaven or down to hell.

—Exchange.

### COTTON AND CORN.

Coten am de King,
But con am de Queen,
So plant er row ob hit,
Een ber tween.

Cotin am de crap
Dat brings de cash
But con am de one
Dat gibs de mash.

So plant bof er dem,
Wid corn ber tween,
De King kaint lib,
Wid dout 's Queen.

—B. H. Teague.

